# Something about "Germs." The Views of Professor Charteris of the Glasgow University

**Published:** 1888-12-22

**Authors:** 


### Something About "Germs."

### The Views of Professor Charteris, of the Glasgow University.

In calling anything by the name of "germ" we put forward a large assumption. A "germ" implies "germinal" power, the power of development; the power of increase—reproduction; the power of influencing surrounding circumstances. The idea of germs in connexion with disease is not pleasant; it is, in fact, rather horrifying. Why should any Creator have created plants or animals whose sole function seems to be to produce in man and other mammals painful disease, disability for work, and sometimes death? The question is difficult to answer on any philosophic analysis of life and the universe. The doctrine of development helps us no more than the older theory of independent creative acts. It seems to be pretty certain that some germs do produce certain diseases; that the same germs under similar circumstances produce the same diseases; that, in fact, one end in life which these creatures have to serve is the production of those very diseases; perhaps it is their only end. Here is a nut for both the Evolutionists and the Creationists, if we may coin a word, to crack. We call attention to it for one purpose only, and that is to help both Creationists and Evolutionists to see clearly, as some of them do not seem to see, that they have not got quite to the end of all things in heaven and earth yet, either in their theories or in their facts. The subject has wide-reaching interests. It is by no means merely a question for medical men or "health prophets." The philosopher and the divine will both be the better for a clear insight into its difficulties, and a full appreciation of its philosophical significance.

Professor Charteris, of the Glasgow University, expounded his views on germs to the assembled students a few weeks ago. Speaking to medical students, he discussed the subject under medical limitations. It has been the fashion among medical men to prefer a single key for the opening of all possible doors. They like a leading principle which will explain all diseases. There have been several such principles during the ages. Doctors know of the "solidists," the humoralists, and various other "ists." The "germists" are now in vogue. There is nothing surprising in all this. The clergy have always done exactly the same. They love to condense all religion into a single essential principle. One puts forward his "church," another his "book," a third his "creed." They are all more or less anxiously searching for simplicity, but above all for "unity," as Sir William Hamilton says. They seek the "one" in "the many:" the "one" which will explain "the many;" the "one" which in a truly philosophic sense *is* "the many." Whatever may be thought of the skill or of the success of the seekers at any given period, no one can question the value of their object or the merit of their good intention. The aim and the intention are as old as man; they are the fruit of the human tree, the necessary result of the operations of that faculty called "mind" which no creature on earth, except man, possesses. Man being man must think; man being man must philosophise; man being man must expound and explain.

Dr. Charteris expounds "germs" and their doings as he understands them at the present moment. Has he hit upon the exact facts? Does he comprehend all and everything about them? We do not know: neither does he. What he sees under the microscope, that he knows something about: what results he produces by his cultivations, these he has some limited knowledge of. Almost everything outside these limits is more or less ingenious speculation and theorising. Very proper speculation, very honourable theorising—but still to be recognised as "theorising."

What are bacteria?" says the Professor. The answer is very simple. "They are vegetable cells, the lowest members of the vegetable kingdom." That sounds as if it should make an end of all mystery. But what precisely is a "vegetable cell," and how does Professor Charteris know that these little organisms have plumbed the deepest depths of vegetable existence? He does not know. The next statement about these "vegetable cells" is hardly framed with that precision which one likes to see in a rigidly scientific proposition. "They possess," says the doctor, "constancy of form; although they may vary more or less in outward appearance under varying circumstances, yet each species has always a certain definite form marking its complete development." Would it not have been better, Mr. Professor, to have said, "the full-grown bacteria of the same breed always have the same shape?" It would at any rate have been simpler, and the students would probably have both understood and remembered it better.

There are several recognised varieties of germs. Professor Charter is treats of four. The first is round or globular, and is called a "micrococcus;" the second is a short rod, and to it the name of "Bacterium" is properly applied, although all the varieties are often included under "Bacteria" by certain writers; the third is a longer rod, and is named "Bacillus;" the fourth is a twisted spiral, and is designated "Spirochæta." All these organisms have a definite shape, as has been said, when full-grown; but of course in the growing stages they vary a good deal. It is necessary to remember this, otherwise species and varieties will be multiplied in the mind of the observer without the warrant of actual fact. All the varieties have the power of reproduction. Some of them, it seems to be proved, are the definite causes of certain diseases. These always give rise to the same disease, never to any other. Of course they do not always produce the same severity of attack, neither is it known whether or not they infalliby produce disease whenever they are introduced into the human system. If it be certain, as it is, that they act with varying degrees of intensity on different people, it seems probable that on some people they will not act at all; at any rate, unless present in overwhelming numbers and force.

Germs, though so excessively minute, and so very low in the scale of vegetable life, are terrible creatures if all that is said of them be true. It has always been thought a wonderful thing that the walls of Jericho should have fallen down when Joshua's lusty priests blew mighty blasts on their rams' horns. But that wonder was small compared with this, that a small crop of germs in a Newton's or a Gladstone's lung would have put a prompt termination to all their intellectual activity in this sphere. The world is full of miracles, much more wonderful than the rams' horns story. These little creatures multiply at an enormous rate, and develop their destructive power with equal rapidity. It takes from twenty to thirty years to make a fully-developed man. The particular germs which can promptly kill him may be developed in a few days or weeks. It does not seem to be the mere presence of the germs which destroys the human organs in which they grow, but the germs, plus certain chemical products of the nature of alkaloids, which are antagonistic both to the organisms themselves, and to the vital tissues in which they may grow. It is believed that these chemical products excite putrefaction and fermentation, and the consequent destruction of the part involved.

There is one note of consolation in Professor Charteris's rather dismal vaticination. "We can in some degree," says he, "deprive the germs of their noxious influence by changing their environment." That is a ray of light. The Bastile had such thick walls and such secure locks, that obnoxious persons once enclosed within abandoned hope. We need not give up hope about germs. If it be true, as seems most probable, that "the plague," the "black death," cholera, small-pox, scarlet fever, and all such diseases are due to germs, then we have already made much headway in the conquest of their death-bringing kingdom. "Sanitation," that is knowledge and care, have been our victorious generals. "Sanitation" must be broadened and deepened until it includes everything in man's life and environment that affects his physical, mental, and moral well-being. "Sanitation" is an evangel of life; of life victorious over death. Happy is that man and that country which comprehends, believes in, and practises its saving precepts.

